# The protective role of gamma zone peripapillary atrophy in diabetic retinopathy: insights from deep learning and SS-OCT angiography

**DOI:** 10.3389/fcell.2024.1501625

**Published:** 2024-12-04

**Authors:** Yingying Li, Xinxin Hu, Xueqiong Ye, Qingya Zhong, Xixi Zhang, Jinglin Zhu, Jiahui Jiang, Dandan Wang, Juntao Zhang, Weina Ren, Yitian Zhao, Qinkang Lu, Na Zhao

**Affiliations:** ^1^ Department of Ophthalmology, The Affiliated People’s Hospital of Ningbo University, Ningbo, China; ^2^ Ningbo Clinical Research Center for Ophthalmology, Ningbo, China; ^3^ Ningbo Key Laboratory for neuroretinopathy medical research, Ningbo, China; ^4^ Eye Hospital of Wenzhou Medical University (Ningbo Branch), Ningbo, China; ^5^ Laboratory of Advanced Theranostic Materials and Technology, Ningbo Institute of Materials Technology and Engineering, Chinese Academy of Sciences, Ningbo, China

**Keywords:** gamma zone, peripapillary atrophy, diabetic retinopathy, deep learning, optical coherence tomography angiography

## Abstract

**Purpose:**

To explore the relationship between peripapillary atrophy (PPA) and diabetic retinopathy (DR), and to uncover potential mechanisms using swept-source optical coherence tomography (SS-OCT) angiography.

**Methods:**

This cross-sectional study included 845 patients with type 2 diabetes (T2DM), who underwent detailed systemic and ophthalmic evaluations. A state-of-the-art deep learning method was employed to precisely identify the parapapillary beta and gamma zones. Based on PPA characteristics, eyes were categorized into four groups: without beta or gamma zone (Group A), isolated beta zone (Group B), isolated gamma zone (Group C), and with both beta and gamma zone (Group D). Digital fundus photography was utilized to diagnose and stage DR severity, while SS-OCT angiography quantified retinal and choroidal vasculature.

**Results:**

Participants had a mean age of 66 ± 8.8 years, with 437 (51.7%) male. Beta and gamma PPA zones were observed in 574 (67.9%) and 256 (30.3%) eyes, respectively. Beta zone PPA was associated with older age, whereas gamma zone PPA was correlated with longer axial length (AL), lower vessel density, and reduced choroidal thickness. Adjusted analyses revealed that eyes with isolated (Group C) or concurrent (Group D) gamma zone PPA conferred significantly lower DR grade, independent of known risk factors including systemic diabetes risk factors and AL.

**Conclusion:**

This study finds that gamma zone PPA is linked to a reduced risk of developing DR. These results imply that the gamma zone may reflect progressive myopia-associated thinning and microvascular depletion in posterior ocular tissues, potentially contributing to structural resilience against DR. This novel insight offers a promising avenue for understanding the interplay between PPA and DR.

## 1 Introduction

Diabetes mellitus is a major public health issue that projected to affect 693 million adults by 2045 (Cho et al., 2018). Diabetic retinopathy (DR) is a major microvascular complications of diabetes mellitus, occurring in about 30%–40% diabetic patients (Yau et al., 2012). DR has become one of the leading causes of low vision and blindness among working-age adult population (Ting et al., 2016). Recognition of people at higher risk for DR progression is important for preventing visual impairment.

In contrast to established systemic risk factors like duration of diabetes, hyperglycemia, hypertension, hyperlipidemia and obesity, there are few ocular risk factors associated with DR (Ting et al., 2016). Previous studies have demonstrated that myopia and increased axial length (AL) have a protective effect against DR progression (Chao et al., 2016; Lim et al., 2010; Man et al., 2012). The proposed mechanism for this protective effect is that the degenerative lesions caused by long-term fundus stretching may be more resistant to hypoxia and ischemia (Chao et al., 2016; Man et al., 2013). However, compared to AL alone, myopic fundus signs may be more indicative of choroidal thinning and thus may correlate more closely with decreased DR prevalence. On this basis, Tan, et al. enrolled 592 diabetic patients and compared their DR severity between eyes with or without conventional ophthalmoscopic beta zone of peripapillary atrophy (PPA) (Tan et al., 2018). The results showed that eyes with PPA-beta were less likely to have DR, independent of both AL and refractive status.

PPA is the most common myopic fundus change and is characterized by a shift and widening of the Bruch’s Membrane Opening (BMO) and retinal pigment epithelium (RPE) opening. PPA can be differentiated into an αlpha zone and a conventional beta zone based on its clinical appearance (Jonas et al., 1989). The αlpha zone presents with irregular hypopigmentation and hyperpigmentation of the RPE. The conventional beta zone shows exposed sclera and choroidal macrovascular with complete RPE atrophy, located between the optic border and αlpha zone. With the development of spectral-domain optical coherence tomography (SD-OCT), the conventional beta zone has been further categorized into a (new) beta zone and a gamma zone based on the BMO (Dai et al., 2013). Current studies found beta zone associated with older age and the glaucoma prevalence (Kim et al., 2014; Shang et al., 2019; Yamada et al., 2016), while gamma zone related to axial myopia (Jonas et al., 2016; Kim et al., 2013; Zhang et al., 2018). Most previous studies focused on the relationship between conventional beta zone and ocular diseases, with little known about new beta zone and the gamma zone. Therefore, the aim of our study was to investigate whether eyes with new beta or gamma zone involvement, or both, are less likely to have DR, and to explore the potential vascular factors associated with this phenomenon.

## 2 Methods

### 2.1 Study participants

The data were from the Ophthalmology Center of The Affiliated People’s Hospital of Ningbo University. All procedures adhered to the tenets of the Declaration of Helsinki, and ethics approval was obtained from the Ethics Committee of the Ophthalmology Center of The Affiliated People’s Hospital of Ningbo University, China. Written informed consents were obtained from all participants.

A total of 845 participants with type 2 diabetes mellitus (T2DM) were enrolled in our study. The inclusion criteria were: (1) prior T2DM diagnosis using the WHO diagnostic criteria. (2) intraocular pressure (IOP) ≤ 21 mmHg in both eyes. The exclusion criteria were: (1) presence of glaucoma, optic neuropathy or systemic diseases affecting ocular circulation; (2) cataract surgery or other intraocular surgery within 3 months pre-enrollment; (3) low quality of OCTA, structural OCT, or color fundus images; (4) inability to cooperate with the examination. If both eyes met the eligibility criteria, one eye per participant was randomly selected.

### 2.2 General and laboratory examinations

A standardized interview, consisting of predetermined questions, was conducted with all participants to gather relevant information such as age, sex, date of birth, general medical history, lifestyle factors, and history of eye disease and surgery. Additionally, systolic blood pressure (SBP) and diastolic blood pressure monitor (DBP) were measured using a blood pressure monitor (HBP-9030; Omron, Kyoto, Japan). The mean arterial pressure (MAP) was determined by the formula: MAP = 1/3SBP + 2/3DBP. Height and weight were measured by the general practitioners. Body mass index (BMI) was then calculated by dividing weight by height in meters squared (kg/m^2^). Furthermore, venous blood samples were collected from the subjects to measure fasting plasma glucose (FPG), glycosylated hemoglobin (HbA1c), urine albumin creatine ratio (UACR), total cholesterol (TC), low-density lipoprotein (LDL), high-density lipoprotein (HDL), and triglycerides (TG).

### 2.3 Ocular examination

A comprehensive ophthalmic examination was conducted on all participants by the same experienced operator between 8:00 a.m. and 11:30 a.m. each day. IOP was measured using a noncontact tonometer (Auto Tonometer TX-F; Topcon, Tokyo, Japan). The anterior and posterior segments of participants were examined using slit-lamp biomicroscopy (SL130, Zeiss, Jena, Germany). Additionally, the axial length (AL) was measured using IOLMaster (Carl Zeiss Meditec AG, Jena, Germany). Refractive error was excluded from the study due to a significant number of eyes having undergone prior cataract surgery.

### 2.4 Deep learning algorithm for determining PPA

**FIGURE 1:**
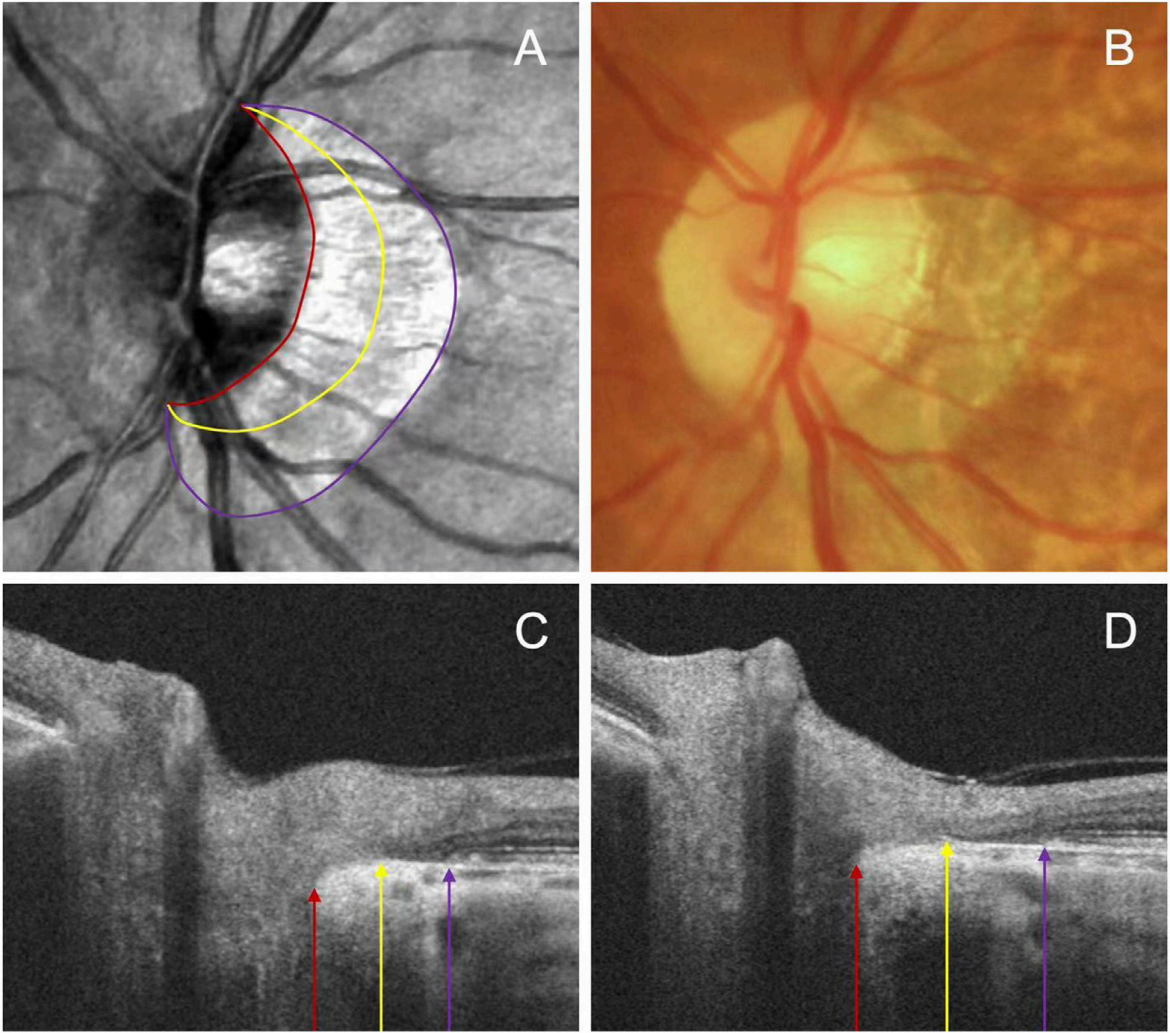
Representative images illustrating the subzones of PPA. The OCT images **(A)** and fundus photography **(B)** are centered on the same position. The beta zone between the purple and yellow arrow, and gamma zone between the yellow and red arrow on the serial B-scan images **(C, D)**.

To assess peripapillary atrophy (PPA), we employed swept-source OCT (SS-OCT, BM-400K BMizar, TowardPi Medical Technology Co., Ltd., Beijing, China) and examined its characteristics on B-Scan. The beta zone was identified by the presence of BM without the RPE, whereas the gamma zone PPA was noted by the absence of BM, situated between the optic disc margin and the beta zone (Figure 1). We classified eyes into four categories: those without beta or gamma zone (Group A), those with only a beta zone (Group B), those with only a gamma zone (Group C), and those with both beta and gamma zone (Group D).

To delineate the conventional beta zone PPA, we utilized an innovative image segmentation algorithm, nnU-Net (Isensee et al., 2021), to precisely define the optic disc boundary and RPE atrophy on *en face* images. A dataset consisting of 200 manually annotated *en face* images was employed for the training and validation of our model. The dataset was partitioned into training and validation subsets in a 7:3 ratio. We selected the Stochastic Gradient Descent (SGD) with Nesterov momentum set to 0.99 as our optimizer, and initialized the learning rate at 0.001 for our experiments. The training process spanned 1,000 epochs, with each epoch containing 250 iterations. The segmentation results demonstrated robustness and effectiveness, achieving a Dice coefficient exceeding 0.95. Eyes lacking the conventional beta zone PPA were classified into Group A (without beta or gamma zone), were distributed among the remaining three groups based on the location of the Bruch’s membrane opening (BMO).

For precise detection of the BMO on B-scan series, we utilized the cutting-edge YoLo-V8 algorithm (Wang et al., 2024). This sophisticated object detection model is celebrated for its real-time processing of high-resolution images and its extensive application in medical image detection, ensuring accurate target identification. We adopted the Stochastic Gradient Descent (SGD) with a Nesterov momentum of 0.937 as the optimizer, with an initial learning rate of 0.001. The training consisted of a maximum of 300 epochs. The model was trained and validated using data from 20 subjects, incorporating 2,500 manually annotated B-scan images, with its reliability well-documented in prior studies (Li et al., 2024). If the BMO coincided with the optic disc border, indicating an absence of the gamma zone, the eyes were assigned to Group B (isolated beta zone). Conversely, if the BMO was located between the optic disc margin and RPE atrophy, the eyes were assigned to Group D (both beta and gamma zones). A minority of eyes displayed no new beta zone, with their BMO coinciding with the RPE atrophy border, thereby categorizing them into Group C (isolated gamma zone). Any discrepancies were adjudicated by a senior third reviewer (ZN).

### 2.5 Assessment of DR

Digital fundus photography (Canon CR-2; Tokyo, Japan) was employed for the diagnosis and staging of DR severity. The fundus camera captured two images, one centered on the macula and the other on the optic disc. In cases where the lesions were unclear, SS-OCT angiography was utilized. SS-OCTA employed a fovea-centered 12 mm × 12 mm scan pattern with an A-scan rate of 400 KHz and an axial scan depth of 6 mm, allowing for more precise identification of the lesions. After a qualitative assessment, two trained ophthalmologists (LY and XH) independently graded DR according to the International Clinical DR Disease Severity Scale (Wilkinson et al., 2003). In case of any uncertainties, a senior ophthalmologist (ZN) would reassess the images.

### 2.6 SS-OCT angiography imaging acquisition and analysis

The SS-OCT angiography system automatically segmented the retina and choroid into sublayers for measurement of microstructural parameters. In this newly developed system, the retina and choroid were automatically stratified into several sub-layers using built-in custom segmentation. The retinal layers were divided into the superficial retinal layer (from the inner limiting membrane (ILM) to the inner plexiform layer (IPL)) and the deep retinal layer (from IPL to the outer plexiform layer (OPL)). The choroid was segmented into the choriocapillaris (CC, from the BM to 29 μm below it) and the large medium-sized choroidal vessels (LMCV, from 29 μm below BM to the choroidoscleral interface (CSI)). Images with graded ≥8 were included. The primary data, including the average vessel density and thickness of the superficial capillary plexus (SCP), deep capillary plexus (DCP), choriocapillaris (CC), and large medium-sized choroidal vessels (LMCV) at a scale of 12 mm × 12 mm, were measured using the built-in software.

### 2.7 Statistical analysis

Statistical analyses were conducted using SPSS version 29.0 (SPSS, Chicago, IL, United States). Continuous data are presented as the mean (standard deviation), while categorical data are presented as the number (%). Group differences in continuous metrics were assessed by one-way analysis of variance (ANOVA) with Bonferroni correction. And chi-square tests compared categorical variables. Multivariable logistic regression examined associations of PPA and other risk factors with DR severity, adjusting for potential confounders. Given the influence of age on beta zone PPA, a subgroup analysis stratifying by age (≤60 years or >60 years) was also performed. All *P*-values were bilateral, and statistical significance was determined when the *P*-values were lower than 0.05.

## 3 Results

This cross-sectional study was conducted between April to June 2023, involving a total of 845 eyes from 845 T2DM participants who were recruited for our study. Among them, 408 (48.3%) were male, and 437 (51.7%) were female. Their mean age (standard deviation) was 66 (8.8) years; Out of the total eyes, 437 (51.7%) were found to have DR. The beta zone PPA was present in 574 eyes (67.9%), while the gamma zone PPA was found in 256 (30.3%).

### 3.1 Comparison of demographic and clinical characteristics among four groups

All participants were categorized based on PPA status into: 198 eyes without beta or gamma zone were classified into Group A, 391 eyes with isolated beta zone were classified into Group B, 73 eyes with isolated gamma zone were classified into Group C, 183 eyes with both beta and gamma zone were classified into Group D. Demographic and clinical characteristics among four groups were summarized in Table 1. No significant difference was observed among the groups in terms of sex, waist, BMI, hypertension, hyperlipidemia, DBP, SBP, MAP, UACR, TC, LDL, HDL, TG, and IOP. However, it was noted that patients in Group B and Group D with beta zone PPA were more likely to be older. Moreover, Group C and Group D had significantly longer AL when compared to Group A and Group B (*P* < 0.001). Additionally, compared to Group A, the other three groups exhibited lower FPG, HbA1c and DR grade (*P* = 0.037, *P* = 0.037, and *P* = 0.001, respectively).

**TABLE 1:** Comparison of demographic and clinical characteristics among four groups.

Variables	Group A (n = 198) Without beta or gamma zone	Group B (n = 391) Isolated beta zone	Group C (n = 73) Isolated gamma zone	Group D (n = 183) Both beta and gamma zone	*P* value
Male, gender, %	88 (44.4)	195 (49.9)	27 (37.0)	98 (53.6)	0.06
Age, years	64.0 (7.4)	66.9 (8.3)	63.7 (9.5)	67.2 (10.5)	**<0.001**
Waist, cm	86.4 (9.0)	86.1 (9.1)	86.0 (8.1)	85.3 (9.1)	0.69
BMI, kg/m^2^	24.6 (3.2)	24.7 (3.3)	25.0 (3.1)	24.3 (3.2)	0.46
Hypertension, %	114 (60.6)	236 (63.4)	39 (58.2)	107 (63.3)	0.81
Hyperlipidemia, %	65 (34.6)	126 (33.9)	23 (34.3)	57 (33.7)	0.10
DBP, mmHg	78.4 (9.6)	78.7 (9.3)	78.7 (8.0)	78.3 (8.7)	0.97
SBP, mmHg	137.6 (16.8)	139.6 (18.0)	137.1 (15.5)	139.6 (17.6)	0.47
MAP, mmHg	98.2 (10.9)	99.0 (10.8)	98.2 (9.4)	98.7 (10.4)	0.82
FPG, mmol/L	7.7 (2.4)	7.4 (1.9)	7.2 (1.8)	7.1 (1.9)	**0.037**
HbA1c, %	7.2 (1.5)	7.0 (1.3)	6.8 (1.0)	6.9 (1.2)	**0.037**
UACR, mg/g	108.7 (474.0)	73.1 (238.2)	67.3 (160.6)	61.9 (159.4)	0.45
TC, mmol/L	4.8 (1.2)	4.7 (1.2)	4.7 (1.2)	4.7 (1.0)	0.89
TG, mmol/L	1.6 (1.0)	1.7 (1.1)	1.8 (1.7)	1.7 (1.0)	0.49
HDL, mmol/L	1.3 (0.3)	1.3 (0.3)	1.3 (0.3)	1.3 (0.3)	0.61
LDL, mmol/L	2.6 (0.8)	2.4 (0.8)	2.6 (0.8)	2.4 (0.7)	0.15
AL, mm	23.1 (0.9)	23.2 (0.8)	24.0 (1.3)	24.3 (1.5)	**<0.001**
IOP, mmHg	14.3 (3.0)	14.2 (3.1)	14.8 (2.2)	14.1 (3.0)	0.46
DR, %	137 (69.2)	214 (54.7)	19 (26.0)	67 (36.6)	**<0.001**
Average DR grade	1.1 (1.0)	0.8 (0.9)	0.3 (0.7)	0.6 (0.9)	**<0.001**

BMI = body mass index; DBP = diastolic blood pressure; SBP = systolic blood pressure; MAP = mean arterial pressure; FPG = fasting plasma glucose; HbA1c = glycosylated hemoglobin; UACR = urine albumin creatine ratio; TC = total cholesterol; TG = triglycerides; HDL = high-density lipoprotein; LDL = low-density lipoprotein; AL = axial length; IOP , intraocular pressure; DR = diabetic retinopathy.

Continuous data are presented as the mean (standard deviation); Categorical data are presented as the number (%).

Factors with statistical significance are shown in boldface.

### 3.2 Associations of risk factors with DR

Associations between DR and systemic factors, AL, and PPA were assessed by logistic regression (Table 2). In the fully adjusted model, higher HbA1c and UACR were found to be associated with increased odds of DR (*P* < 0.001 and *P* = 0.039, respectively). While FPG exhibited a positive association with DR in the univariable model (*P* < 0.001), but this relationship was attenuated after controlling for confounding variables in the multivariable model (*P* = 0.47). Similarly, AL displayed a negative association with DR in the univariable model (*P* = 0.027), but this association became non-significant in the multivariable model (*P* = 0.89).

**TABLE 2:** Associations of risk factors with DR grade.

	Univariable model			Multivariable model		
Variables	Β	Or (95% *CI*)	*P* value	β	Or (95% *CI*)	*P* value
FPG, mmol/L	0.252	1.287 (1.206,1.373)	**<0.001**	−0.050	0.952 (0.856, 1.057)	0.47
HbA1c, %	0.575	1.777 (1.596, 1.978)	**<0.001**	0.582	1.019 (1.010, 1.028)	**<0.001**
UACR, mg/g	0.001	1.001 (1.001, 1.001)	**<0.001**	0.001	1.001 (1.000,1.001)	**0.039**
AL, mm	−0.139	0.870 (0.770, 0.984)	**0.027**	0.011	1.011 (0.867, 1.178)	0.89
PPA						
Group A	Reference			Reference		
Group B	−0.465	0.628 (0.458, 0.861)	**0.004**	−0.429	0.651 (0.450, 0.941)	**0.022**
Group C	−1.724	0.178 (0.100, 0.319)	**<0.001**	−1.611	0.200 (0.099, 0.404)	**<0.001**
Group D	−1.180	0.307 (0.208, 0.454)	**<0.001**	−1.055	0.348 (0.214, 0.568)	**<0.001**

FPG = fasting plasma glucose; HbA1c = glycosylated hemoglobin; UACR = urine albumin creatine ratio; AL = axial length; PPA peripapillary atrophy; Group A = without beta or gamma zone; Group B = isolated beta zone; Group C = isolated gamma zone; Group D = with both beta and gamma zone.

Multivariable model adjusted for age, gender, SBP, FPG, HbA1c, UACR, and AL.

Factors with statistical significance are shown in boldface.

Regarding PPA, individuals with isolated beta zone (Group B) showed lower odds of developing DR compared to those without beta or gamma zone (Group A) (OR 0.651, 95% confidence interval [*CI*] 0.450–0.941, *P* = 0.022). Moreover, individuals with isolated gamma zone (Group C) and those with both beta and gamma zone (Group D) demonstrated additional protective effects against DR (OR 0.200, 95% *CI* 0.099–0.404, *P* < 0.001; and OR 0.348, 95% *CI* 0.214–0.568, *P* < 0.001; respectively). It should be noted that beta zone PPA is known to be associated with aging, and thus, we conducted a subgroup analysis based on age (≤60 years or >60 years), as shown in Table 3. Notably, the association between isolated beta zone PPA (Group B) and DR was no longer significant in this subgroup analysis.

**TABLE 3:** Subgroup analysis of four groups with DR by age (≤60 years or >60 years).

	Multivariable model		
Variables	β	Or (95% *CI*)	*P* value
≤60 years			
Group A (without beta or gamma zone)	Reference		
Group B (isolated beta zone)	−0.449	0.639 (0.329, 1.240)	0.19
Group C (isolated gamma zone)	−2.302	0.100 (0.029, 0.342)	**<0.001**
Group D (with both beta and gamma zone)	−1.649	0.192 (0.071, 0.522)	**0.001**
>60 years			
Group A (without beta or gamma zone)	Reference		
Group B (isolated beta zone)	−0.376	0.686 (0.440, 1.070)	0.10
Group C (isolated gamma zone)	−1.350	0.259 (0.109, 0.618)	**0.002**
Group D (with both beta and gamma zone)	−0.879	0.415 (0.234, 0.736)	**0.003**

Multivariable model adjusted for age, gender, SBP, FPG, HbA1c, UACR, and AL.

Factors with statistical significance are shown in boldface.

Considering the closer relationship that should exist between different groups (mainly the gamma zone) and axial length growth, we conducted a further subgroup analysis based on axial length classification. We conducted a subgroup analysis based on axial length (≤24 mm or >24 mm) (Table 4). For eyes with an AL of less than 24 mm, individuals with an isolated beta zone (Group B) showed a reduced likelihood of developing DR compared to those without beta or gamma zones (Group A) (OR 0.604, 95% *CI* 0.407–0.896, *P* = 0.012). Additionally, individuals with an isolated gamma zone (Group C) and those with both beta and gamma zones (Group D) exhibited further protective effects against DR (OR 0.252, 95% *CI* 0.108–0.587, *P* = 0.001; and OR 0.363, 95% *CI* 0.199–0.661, *P* = 0.001; respectively). For eyes with an AL greater than 24 mm, those with an isolated gamma zone (Group C) and those with both beta and gamma zones (Group D) demonstrated protective effects against DR compared to Group A (OR 0.113, 95% *CI* 0.027–0.477, *P* = 0.001; and OR 0.279, 95% *CI* 0.094–0.832, *P* = 0.022; respectively). Notably, the association between isolated beta zone PPA (Group B) and DR was not significant in this subgroup analysis, consistent with our previous findings. Furthermore, our subgroup analyses indicated that the protective association of gamma zone PPA (Groups C and D) against DR was more pronounced in eyes with longer AL (>24 mm). These results further confirm the protective effect of gamma zone PPA on DR is not solely due to AL.

**TABLE 4:** Subgroup analysis of four groups with DR by AL (≤24 mm or >24 mm).

	Multivariable model		
Variables	β	Or (95% *CI*)	*P* value
≤24 mm			
Group A (without beta or gamma zone)	Reference		
Group B (isolated beta zone)	−0.504	0.604 (0.407, 0.896)	**0.012**
Group C (isolated gamma zone)	−1.379	0.252 (0.108, 0.587)	**0.001**
Group D (with both beta and gamma zone)	−1.014	0.363 (0.199, 0.661)	**0.001**
>24 mm			
Group A (without beta or gamma zone)	Reference		
Group B (isolated beta zone)	−0.214	0.807 (0.264, 2.472)	0.707
Group C (isolated gamma zone)	−2.180	0.113 (0.027, 0.477)	**0.003**
Group D (with both beta and gamma zone)	−1.276	0.279 (0.094, 0.832)	**0.022**

Multivariable model adjusted for age, gender, SBP, FPG, HbA1c, UACR, and AL.

Factors with statistical significance are shown in boldface.

### 3.3 Comparison of microstructural characteristics among four groups

**FIGURE 2:**
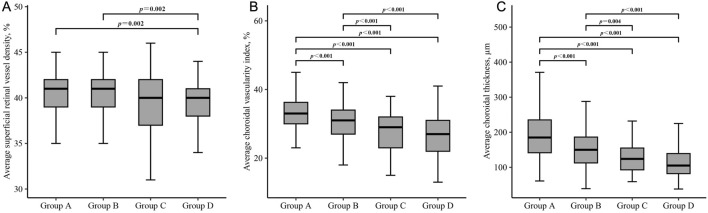
Box plots showing the comparison of superficial retinal vessel density **(A)**, choroidal vascularity index **(B)** and choroidal thickness **(C)** in each group. Group A = without beta or gamma zone; Group B = isolated beta zone; Group C = isolated gamma zone; Group D = with both beta and gamma zone. Significance was set at *P* < 0.00833 for multiple testing in Bonferroni correction.

Significant differences between-group differences were observed in superficial retina, deep retina, and choroid metrics (Table 5). The average superficial retinal vessel density for each group was as follows: Group A - 40.5% (2.2), Group B - 40.4% (2.3), Group C - 39.4% (3.3), and Group D - 39.5% (2.6). Notably, individuals with no beta or gamma zone (Group A) and those with isolated beta zone (Group B) exhibited higher superficial retinal vessel density compared to individuals with both beta and gamma zone (Group D) (all *P* = 0.002) (Figure 2A).

**TABLE 5:** Comparison of microstructural characteristics among four groups.

Variables	Group A (n = 198) Without beta or gamma zone	Group B (n = 391) Isolated beta zone	Group C (n = 73) Isolated gamma zone	Group D (n = 183) Both beta and gamma zone	*P* value
Superficial retina					
Average vessel density, %	40.5 (2.2)	40.4 (2.4)	39.4 (3.3)	39.5 (2.6)	**<0.001**
Average thickness, μm	104.5 (6.9)	103.7 (7.0)	102.7 (6.4)	102.3 (6.5)	**0.021**
Deep retina					
Average vessel density, %	42.3 (2.1)	42.3 (2.3)	42.0 (3.4)	41.7 (2.3)	**0.039**
Average thickness, μm	51.5 (2.9)	51.3 (2.8)	50.8 (2.8)	50.6 (2.5)	**0.020**
Choroid					
Choroidal capillaries, %	47.7 (1.0)	47.8 (1.1)	47.9 (1.2)	47.8 (1.1)	0.82
Choroidal vascularity index, %	33.2 (5.0)	30.6 (4.9)	27.9 (5.9)	26.5 (5.9)	**<0.001**
Average thickness, μm	192.0 (69.0)	153.8 (54.1)	127.3 (48.0)	113.3 (48.0)	**<0.001**

Data are presented as the mean (standard deviation).

Factors with statistical significance are shown in boldface.

Regarding the choroidal metrics, the average choroidal vascularity index (CVI) across the four groups was as follows: Group A - 33.2% (5.0), Group B - 30.6% (4.9), Group C - 27.9% (5.9), and Group D - 26.5% (5.9). Individuals with no beta or gamma zone (Group A) showed the highest CVI, while those with both beta and gamma zone PPA (Group D) demonstrated the lowest CVI among the groups (*P* < 0.001) (Figure 2B). Similar statistically significant differences were observed in choroidal thickness (CT) (Figure 2C). The average CT for each group was as follows: Group A - 192.0 μm (69.0), Group B - 153.8 μm (54.1), Group C - 127.3 μm (48.0), and Group D - 113.3 μm (48.0). Individuals with no beta or gamma zone (Group A) displayed the thickest CVI among four groups (*P* < 0.001). However, there was no significant difference in any microstructural measurement between individuals with isolated gamma zone (Group C) and those with both beta and gamma zone PPA (Group D).

## 4 Discussion

To our knowledge, this is the first study investigating the associations between the beta and gamma zone PPA and DR, including analysis of retinal and choroidal microstructural changes on SS-OCTA. Our study found that presence of PPA, particularly gamma zone involvement, correlated with lower DR odds. Eyes with gamma zone PPA demonstrated reduced retinal and choroidal vascularity along with thinning of choroidal tissues.

The beta zone PPA is defined as the absence of RPE with continued presence of BM. Kim, et al. reported that the PPA with intact BM (defined as beta zone PPA in this study) was associated with older age, theorizing it represents an age-related atrophic change (Kim et al., 2013). Shang, et al. also found a positive correlation between the beta zone area and age (standardized coefficient beta = 0.3, *P* = 0.001) (Shang et al., 2019). Consistent with these findings, our data also demonstrated a higher prevalence of beta zone PPA among older individuals. This may stem from age-related RPE apoptosis and choriocapillaris atrophy (Kim et al., 2013; Lin et al., 2022). We also found no significant difference in axial length (AL) between eyes with or without beta zone PPA, aligning with previous studies suggesting that beta zone PPA is unrelated to axial myopia. Interestingly, our study further revealed that beta zone PPA was not associated with DR odds, while previous studies have found conventional PPA-beta correlated with lower DR odds. The conventional PPA-beta is characterized by exposed sclera and choroidal macrovascular with complete RPE atrophy (Jonas et al., 1989). Previous studies have suggested that development of PPA-beta is associated with axial elongation, leading to optic deviation and directional stretching of the retina and peripheral areas, especially in the temporal section (Jonas et al., 1989; Nakazawa et al., 2008). Tan, et al. reported that individuals with PPA-beta had a lower odds ratio of DR compared to those without (Tan et al., 2018). Additionally, Lin, et al. found differential effects of hyperglycemia on the optic nerve head between type 1 and type 2 diabetes (T1DM, T2DM) without DR (Lin et al., 2022). Specifically, children with T1DM exhibited a significantly smaller area of PPA-beta, while adults with T2DM demonstrated higher optic disc ovality (ODO) compared to corresponding healthy controls. However, as previously mentioned, conventional PPA-beta comprises both new beta zone and gamma zone, which are associated with different ophthalmic diseases. Determining whether the beta zone, gamma zone, or both confer protection against DR was a key goal of our study.

The gamma zone PPA is defined by absence of BM between the beta zone PPA and optic disc. The mechanism behind the formation and enlargement of the gamma zone can be partly explained by the disproportionate growth between retinal structures and the sclera during axial elongation (Chui et al., 2011; Lee et al., 2018). Hayashi, et al. classified PPA in to three categories based on the BM configuration: straight-BM, curved-BM, and defect-BM (Hayashi et al., 2012). Their results indicated a close association between defect-BM and myopia. Similarly, Kim, et al. found that eyes with discontinuous BM (defined as with both beta and gamma zone PPA (Group D) in this study) or lacking BM (defined as isolated gamma zone (Group C) in this study) were associated with longer AL (Kim et al., 2013). Furthermore, they demonstrated a positive correlation between AL and the width of the gamma zone. Our data aligns with these prior studies linking gamma zone PPA to axial myopia. We add evidence that the gamma zone correlated with lower DR odds. This is a novel finding that has not been previously investigated by other researchers, highlighting the originality of our research.

Several mechanisms have been proposed to clarify the protective effect of axial myopia against DR. Lim, et al. found that myopic refractive error and longer AL were associated with longer and narrower retinal arterioles and venules, less tortuous arterioles, lower retinal blood flow, and consequently, decrease retinal capillary pressure and leakage in DR (Lim et al., 2011). Man, et al. proposed that longer AL results in decreased retinal function and oxygen consumption in the outer retina (Man et al., 2013). The decreased metabolic requirement and photoreceptor density in a stretched retina may help to alleviate the effects of hypoxia in DR, thereby reducing the production of inflammatory or proangiogenic cytokines such as vascular endothelial growth factor (VEGF) (Jonas et al., 2009; Ting et al., 2016). Additionally, an elongated eye with increased ocular volume may lead to dilution of VEGF and increased incidence of posterior vitreous detachment (PVD), which served as the vitreous scaffold for neovascular proliferation (Akiba et al., 1990; Yamamoto et al., 2005). The results of our study on microstructural characteristics also demonstrated that eyes with gamma zone PPA were associated with lower vessel density and thinner thickness of choroid. This suggest that the gamma zone is a sign of fundus stretching and thinning, partially explaining its protective mechanism against DR. Interestingly, we observed that individuals without gamma zone PPA showed higher FPG and HbA1c levels, which may lead to choroidal thickening in the early stage of DR (Wang et al., 2020). We hypothesize that hyperglycemia may increase peripapillary blood flow early on, inhibiting ischemic atrophic changes to Bruch’s membrane (BM) and slowing gamma zone progression. Further research should investigate peripapillary microvascular parameters and associated metabolic biomarkers around the beta and gamma zones in diabetic patients. Delineating the impact of glycemic control on peripapillary tissue remodeling may clarify DR changes and inform future interventions to modulate myopia’s protective effects against retinopathy advancement.

This study has several limitations that should be acknowledged. First, the cross-sectional design prevents assessment of DR progression over time in eyes with beta and gamma zone PPA. Second, those with isolated gamma zone changes in DR represented a relatively small subgroup, raising potential for statistical bias. Third, we lacked quantitatively determine the microstructure and microcirculation of PPA subzones, limiting our exploration of the full protective effect of the gamma zone against DR. Thus, the mechanisms underlying gamma zone-mediated protection against DR are incompletely defined. Further research should delineate impacts of gamma zone microvascular remodeling on DR-related pathology. Additional limitations include lack of data on insulin dependence, and other systemic factors that may influence DR risk profiles. Longitudinal follow-up is necessary to clarify causal relationships between PPA subtype progression patterns and incident DR. Larger patient cohorts could also increase power for subanalyses. Nonetheless, our study provides initial evidence to motivate further investigation on the gamma zone’s structural influence in deterring vision-threatening diabetic complications.

In conclusion, we found that gamma zone PPA associated significantly with reduced odds of DR, independent of known systemic risk factors and axial length. The gamma zone seems to represent progressive myopia-driven stretching and thinning of posterior ocular tissues. These structural changes may underpin myopia’s protective effects, conferring resilience of the peripapillary retina and choroid to microvascular complication in diabetes. Further investigation is warranted to confirm the gamma zone’s influence on DR risk and elucidate the intricate relationship between progressive myopia remodeling and development of retinal vasculopathy. Advancing our understanding of structural factors governing susceptibility versus resistance to retinopathy may unveil new therapeutic targets for intervention. Mapping pathological processes from the earliest asymptomatic stages is key to unveiling new strategies to deter vision loss for millions with diabetic eye disease.

## Data Availability

The original contributions presented in the study are included in the article/Supplementary Material, further inquiries can be directed to the corresponding authors.

## Supplementary material

Supplementary Figure 1 Representative images showcasing eyes without the conventional beta zone PPA. The optic border is outlined by a red line and red arrow **(A, C, D)**.

Supplementary Figure 2 Representative images depicting eyes with an isolated new beta zone PPA. The new beta zone is located between the red and purple arrows on the serial B-scan images **(C, D)**.

Supplementary Figure 3 Representative images illustrating eyes with an isolated gamma zone PPA. The gamma zone is demarcated between the red and yellow arrows on the serial B-scan images **(C, D)**.
