# Supplementary figures and images for: The protective role of gamma zone peripapillary atrophy in diabetic retinopathy: insights from deep learning and SS-OCT angiography
Source: Front Cell Dev Biol. 2024 Dec 4;12:1501625. doi: 10.3389/fcell.2024.1501625 (PMC11653018; doi:10.3389/fcell.2024.1501625)

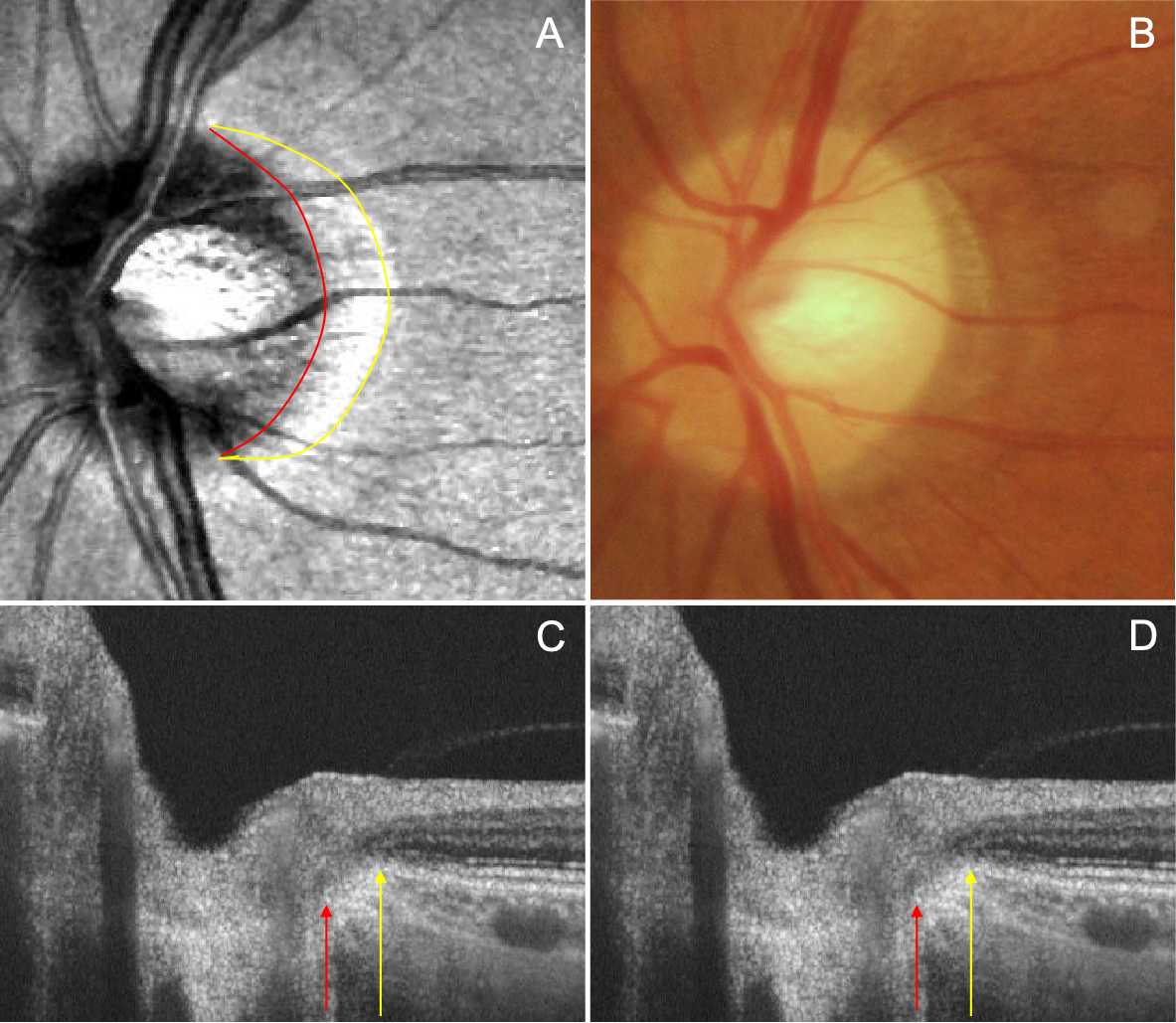

Supplement: Supplementary file 1 [file Image3.jpeg]

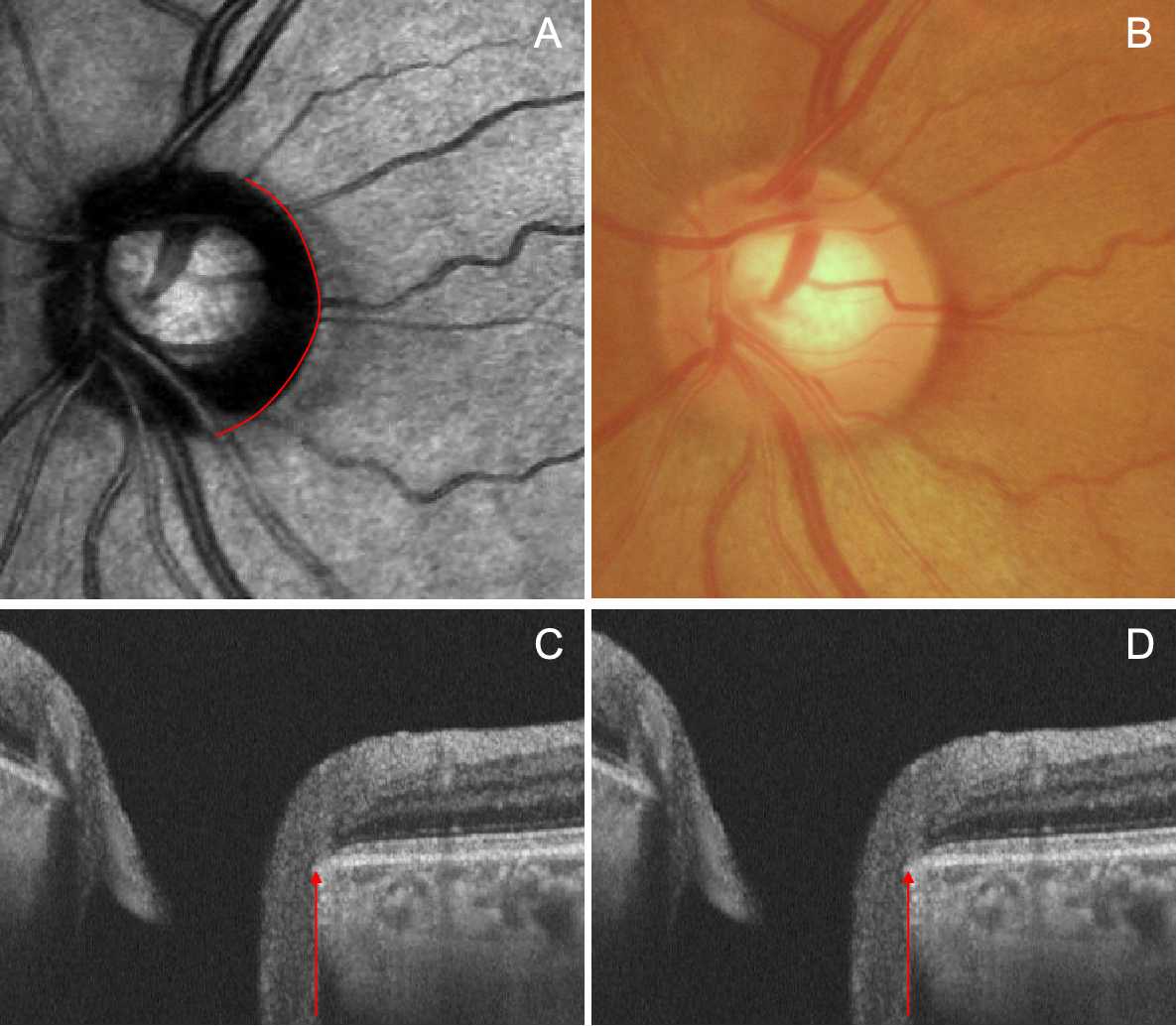

Supplement: Supplementary file 2 [file Image1.jpeg]

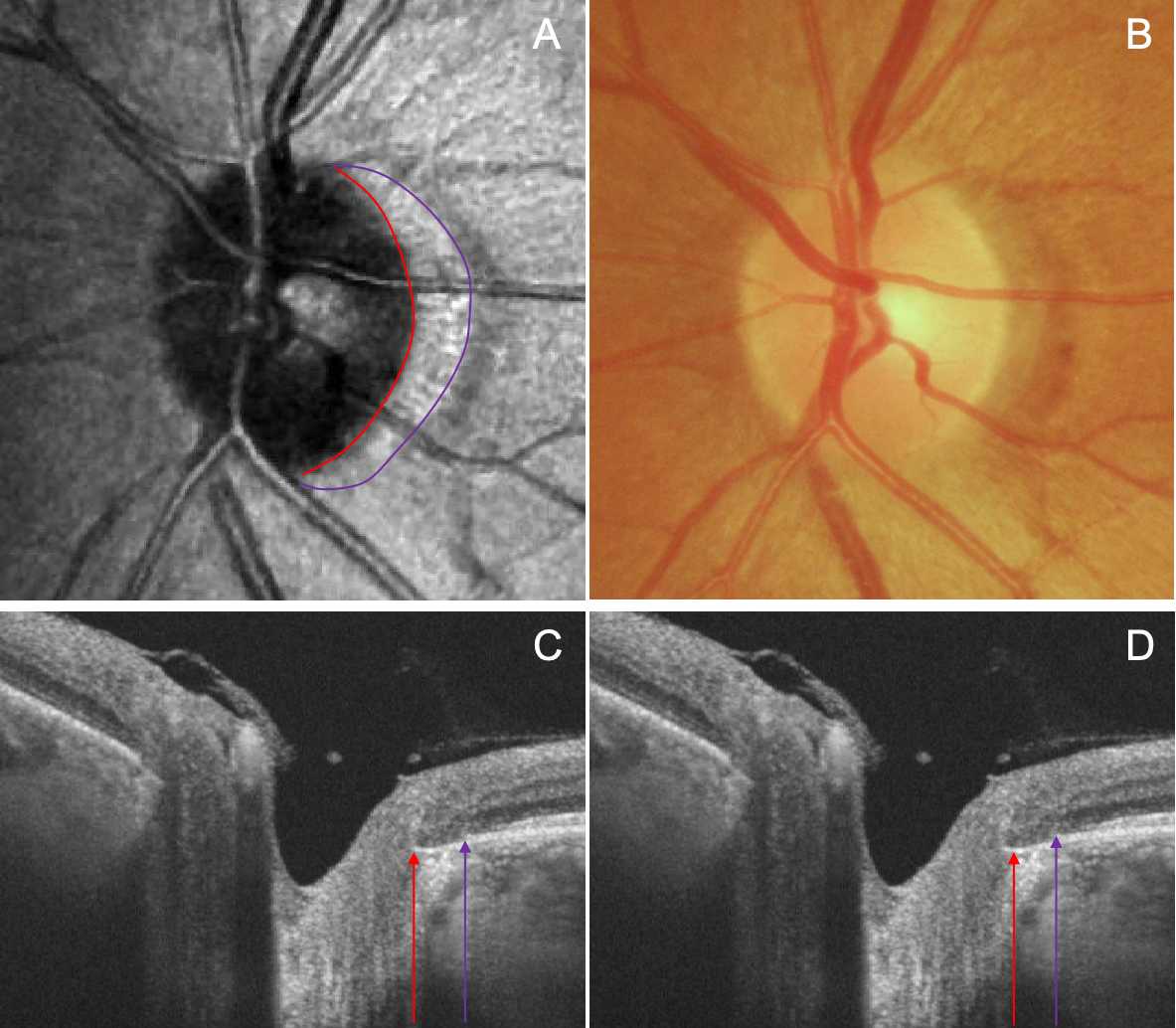

Supplement: Supplementary file 3 [file Image2.jpeg]
